# Effect of pan retinal photocoagulation on central macular thickness and visual acuity in proliferative diabetic retinopathy

**DOI:** 10.12669/pjms.321.8758

**Published:** 2016

**Authors:** Ahsan Mukhtar, Muhammad Saim Khan, Murtaza Junejo, Mazhar Ishaq, Bushra Akbar

**Affiliations:** 1 Dr. Ahsan Mukhtar, FCPS(Ophth), FCPS (VR), FRCS(G), Armed Forces Instituteof Ophthalmology (AFIO), Rawalpindi, Pakistan; 2 Dr. Muhammad Saim Khan, MBBS, Armed Forces Instituteof Ophthalmology (AFIO), Rawalpindi, Pakistan; 3 Dr. Murtaza Junejo, MBBS, Armed Forces Instituteof Ophthalmology (AFIO), Rawalpindi, Pakistan; 4 Prof. Dr. Mazhar Ishaq, FCPS, FRCSEd, FRCOphth, Armed Forces Instituteof Ophthalmology (AFIO), Rawalpindi, Pakistan; 5 Dr. Bushra Akbar, MBBS, Armed Forces Instituteof Ophthalmology (AFIO), Rawalpindi, Pakistan

**Keywords:** Proliferative diabetic retinopathy, Laser pan-retinal photocoagulation, Diabetic macular edema

## Abstract

**Objective:**

To evaluate the effect of pan-retinal photocoagulation with Pattern Scan Laser (pascal)on best corrected visual acuity and central macular thickness in patients having proliferative diabetic retinopathy (PDR).

**Methods:**

This study was conducted at AFIO, Rawalpindi, Pakistan from Oct 2014 to Jul 2015. Sixty seven eyes of 46 patients having proliferative diabetic retinopathy were included in the study. All patients underwent ophthalmic clinical examination including uncorrected distant visual acuity (UCVA), best corrected visual acuity (BCVA), fundus examination with slit lamp and optical coherence tomography to document the pretreatment central macular thickness (CMT). Two sessions of PRP using Pattern Scan Laser were performed 04 weeks apart and OCT was repeated 04 weeks after the 2^nd^ session. Central macular thickness and BCVA were documented.

**Results:**

Sixty seven eyes of 46 patients (29 females and 17 males) with mean age of 57.45 ± 5.78 years underwent treatment with two sessions of laser PRP. Mean pretreatment BCVA was 0.67 ± 0.43 and mean post-treatment BCVA was 0.57 ± 0.3. Mean central macular thickness (CMT)as measured by OCT was 391.93 ± 170.43 before treatment and 316.91 ± 90.42 um after treatment. The magnitude of induced change in CMT after treatment was 75.01 ± 90.75 and BCVA was 0.09 ± 0.14.

**Conclusion:**

Laser PRP with Pattern scan laser alone in patients with combined presentation of PDR and DME is safe and effective.

## INTRODUCTION

Diabetic retinopathy (DR) is the most common blinding microvascular complication of diabetes mellitus and its prevalence is expected to rise significantly over the next 15 years. The worldwide prevalence of diabetes was 6.4% in year 2010 and the expected prevalence is 7.7% by the end of year 2030. Two main reasons for visual loss in diabetic patients are diabetic maculopathy and complications associated with proliferative diabetic retinopathy (PDR).^1^,^2^

The gold standard treatment for PDR is pan-retinal laser photocoagulation (PRP) which is required to be performed soon after detection of retinal neovascularization.^3^ The mechanism of PRPis to convert the ischemic peripheral retina to anoxic state, thus eliminating the ischemic drive for retinal neovascularization and reducing the intravitreal VEGF levels.^4^,^5^

The visual and clinical outcome of patients after PRP is dependent upon the surface area of retina over which the laser is applied.^6^,^7^ Although laser PRP reduces the risk of visual loss in patients with PDR, it may be associated with complications such as visual field loss, macular edema and serious retinal detachment. The incidence of macular edema after PRP has been found in 25% - 43% of the eyes and it is considered to be secondary to retinal inflammation and increased vascular permeability that is triggered by laser PRP, however in long term there is thinning of nerve fibre layer.^8^,^9^,^10^

Initially, Argon blue-green and krypton laser were mainly used for laser photocoagulation in PDR followed by development of diode lasers. However in current clinical practice shorter pulse duration lasers have replaced these lasers due to less cumbersome treatment time and better safety profile.^11^-^13^ Some authors claim that PRP does not induce macular edema especially when using pattern scan laser while others claim short pulse duration treatments are less effective in reducing retinal neovascularization as compare to longer pulse treatment.^14^,^15^

However, these studies included patients who had normal central macular thickness at the time of presentation. The effect of laser PRP on central macular thickness in patients who have some degree of diabetic macular edema (DME) has not been studied so far in our population. The aim of conducting this study was to evaluate the effect of PRP using pattern scan laser (PASCAL) on BCVA and CMT in our population.

## METHODS

This was a Quasi experimental study conducted at Armed Forces Institute of Ophthalmology, Rawalpindi, Pakistan from Oct 2014 to Jul 2015. Open EPI info calculator was used to calculate sample size and it appeared to be 50 eyes. We recruited 76 eyes of 51 patients initially but 5 patients could not have regular follow up and finally we had 67 eyes of 46 patients. Newly diagnosed patients of PDR were included in study except for patients with history of uncontrolled diabetes (HbA1C > 7.0%), uncontrolled hypertension (>140/90) or previous treatment of diabetic retinopathy (intravitreal anti VEGF/laser photocoagulation). Patients having visually significant cataract, taut posterior hyaloid membrane, advanced diabetic eye disease (Pre-retinal / vitreous haemorrhage or tractional retinal detachment)and cystoid macular edema (on OCT) were also excluded from the study. Informed written consent was taken and all the patients underwent ophthalmic clinical examination that included uncorrected distant visual acuity (UCVA), corrected distant visual acuity (CDVA), fundus examination with slit lamp and optical coherence tomography (OCT- Topcon 3D OCT-1 Maestro) to document the pretreatment central macular thickness (CMT). The diagnosis of proliferative diabetic retinopathy was established on clinical examination and fundus fluorescein angiography was performed only in doubtful cases to confirm the diagnosis. Two sessions (04 weeks apart) of laser PRP using PASCAL (Streamline 532 nm Pattern scanning laser) was performed. OCT was repeated 04 weeks after the 2^nd^ session of PRP and CDVA as well as CMT were documented.

### Procedure

We treated the patients with Pattern scanning laser (PASCAL) using Quadraspheric (VOLK) lens. The total number burns at 1^st^ session were approximately 1500 with pulse duration of 20 msec, spot size of 200 um and power ranging from 400 to 500 mW. The burn intensity was titrated until achievement of a mild grey white opacity. PRP was repeated 04 weeks later with an additional 500-1000 burns with same parameters. All sessions were performed by the first author under topical anesthesia with Proparacaine hydrochloride 1%.

### Statistical analysis

Statistical package for social sciences (SPSS 21.0) for windows was used for statistical analysis. The continuous variables in data were described in terms of mean ± SD (standard deviation). The induced change in central macular thickness (CMT) and best corrected visual acuity (BCVA) comparing the preoperative and postoperative measurements were evaluated statistically with paired sample t-test (p ≤ 0.05 significance level).

## RESULTS

Total 67 eyes of 46 patients (29 females and 17males) underwent treatment with two sessions of laser PRP. Age of patients ranged from 48 to 65 years with mean age of 57.45 ± 5.78 years (Table-I). BCVA of patients ranged from 0.17 to 1.77 with mean pretreatment visual acuity of 0.67±0.43 and mean post-treatment visual acuity of 0.57±0.3 (Table-I). The mean number of laser burns were 2313.66±110.78 and the mean power used was 433.96±24.50 mW (Table-I). CMT as measured by OCT was ranging from 174 um to 693um with a mean of 391.93±170.43 before treatment and 193 um to 500 um with a mean of 316.91±90.42um after treatment. The magnitude of induced change in CMT after treatment was 75.01± 90.75 and BCVA was 0.09±0.14 as shown in (Table-II).

**Table-I:** Mean values of various variables.

Variables	N	Min	Max	Mean ± SD
Age	67	48	65	57.45±5.77
Pre treatment BCVA	67	0.17	1.77	0.67±0.43
Pretreatment CMT	67	174	693	391.93±170.43
Post treatment BCVA	67	0.17	1.77	0.57±0.36
Post treatment CMT	67	193	500	316.91±90.42
Number of laser burns	67	2100	2500	2313.66±110.78
Laser Powerused during PRP (mW)	67	400	500	433.96±24.50

**Table-II:** Induced change in visual acuity after treatment.

	Pre treatment	Post treatment	Induced change	P value
CentralMacular thickness (CMT)	391.93±170.43	316.91±90.42	75.01±90.75	P <0.001
Best correctedvisual acuity(BCVA)	0.67±0.43	0.57± 0.36	0.09±0.14	P <0.001

## DISCUSSION

Laser photocoagulation remains the mainstay of treatment for proliferative diabetic retinopathy for the last three decades after Diabetic retinopathy treatment study (DRS) followed by Early treatment diabetic retinopathy study (ETDRS).^16^-^19^ Both of these trials revealed a significant reduction in diabetic retinopathy induced visual loss after laser treatment. Although the exact mechanism of Laser PRP in treatment of PDR is not known but there are several proposed mechanisms. Laser PRP appears to decrease the ischemic drive, abolishing the stimulus for neovascularization and therefore, leads to regression of retinal new vessels. Despite its benefits and effectiveness in PDR, laser photocoagulation of retina is not free of demerits and side effects. Patients after PRP may develop visual field loss, diminished vision secondary to macular edema, serous retinal detachment, epiretinal membrane and macular pucker.^8^,^9^,^20^

Authors have attributed the induced macular edema or increased central macular thickness after PRP to the power and duration of laser treatment. Argon blue green laser, which remained the most commonly used laser type for PRP over decades was associated with increased macular thickness.^13^ Newer short pulse lasers such as Pattern scan laser (PASCAL) with a pulse duration of 20-30 millisecond, which is shorter than conventional Argon laser where the duration is usually 100 millisecond, it was concluded by Muqit et al. that they do not cause macular edema after adequate treatment.^14^,^21^,^22^ However, patients included in these studies were those who did not have macular edema at presentation. We in our study, also included those patients who had increased macular thickness and some amount of macular edema at the time of presentation along with proliferative diabetic retinopathy.

Like Gaucher D et al., in this study we found out that CMT after two sessions (2500-3000 burns) of laser PRP with PASCAL is reduced in statistically significant number of the patients.^23^ We also found a significant improvement in visual acuity. These findings are one step ahead of what has been claimed by Muqit et al. in their study.^21^,^22^ The exact mechanism of this improvement in vision as well as CMT is not known, however it is claimed that reduction of ischemic drive after PRP, reduces the VEGF levels and so the exudation induced by them.^23^ We in our study, included those patients who had better diabetic and hypertensive control so that the confounding effect of hyperglycemia as well as hypertension are negligible.

The short comings of our study were that we did not consider the duration of diabetes, the type and dosage of systemic treatment and the presence of diabetic nephropathy. All these factors can affect the diabetic retinopathy.^24^ Secondly, in our study we observed a relatively short term effect of laser PRP on CMT after an average of 02 sessions 04 week apart before the completion of treatment and complete regression of new vessels.

The combined presentation of PDR with diabetic macular edema (DME) is a common happening and we think our findings are important in such cases. Whether laser PRP with shorter pulse lasers can alone be used as first step of treatment in these patients or it needs to be combined with anti-VEGF agents is still to be evaluated on larger data sets and long term follow up.

## CONCLUSION

We conclude that laser PRP with PASCAL in patients with combined presentation of PDR and DME is safe and effective. It not only reduces the risks and complications associated with progression of neovascularization but also leads to improvement of vision and DME.
